# Efficacy of Insecticides against the Invasive Apricot Aphid, *Myzus mumecola*

**DOI:** 10.3390/insects14090746

**Published:** 2023-09-06

**Authors:** Dania H. Tabet, Edoardo Visentin, Martina Bonadio, Marko Bjeljac, Yazmid Reyes-Domínguez, Andreas Gallmetzer, Urban Spitaler

**Affiliations:** 1 Institute for Plant Health, Laimburg Research Centre, 39040 Auer (Ora), Italy; daniahanna.tabet@laimburg.it (D.H.T.); edoardo.visentin@laimburg.it (E.V.); martina.bonadio@laimburg.it (M.B.); marko.bjeljac@laimburg.it (M.B.); yazmid.reyes-dominguez@laimburg.it (Y.R.-D.); andreas.gallmetzer@laimburg.it (A.G.); 2 Faculty of Science and Technology, Free University of Bozen-Bolzano, 39100 Bozen (Bolzano), Italy

**Keywords:** *Prunus armeniaca*, invasive pest, integrated pest management, pesticides, stone fruit

## Abstract

**Simple Summary:**

The apricot aphid (*Myzus mumecola* Matsumura) is native to Asia and is currently spreading throughout Europe. As this aphid species causes substantial damage to apricot trees, it is important to assess the effectiveness of available insecticides. In this study, we examined 12 different insecticides according to the maximum field dose. To determine the efficacy of each insecticide, aphids were exposed to treated leaf discs of apricot and mortality rates were evaluated at various doses. Additionally, the impact on the colony development was assessed for selected insecticides. Furthermore, we conducted a field trial for insecticides commonly applied in apricot production. In laboratory trials, acetamiprid, deltamethrin, etofenprox, flupyradifurone, pirimicarb, sulfoxaflor, and tau-fluvalinate insecticides resulted in mortality rates ranging from 95% to 100% at the field dose. In the colony development trials, flonicamid, pyrethrins, and spirotetramat exhibited a strong effect. Furthermore, spinosad and azadirachtin demonstrated the least efficacy in reducing the colony development of surviving nymphs per aphid. Based on these findings, it can be concluded that most of the tested insecticides can be used to effectively control *M. mumecola*.

**Abstract:**

The invasive apricot aphid (*Myzus mumecola* Matsumura) is an important pest of apricot trees (*Prunus armeniaca* L.). In the presented study, laboratory bioassays using treated leaf disks of apricot were conducted to test the efficacy of twelve insecticides according to the maximum field dose. Additionally, dose-response curves were established for selected insecticides, and the effects on colony development were evaluated. Furthermore, a field trial was conducted to investigate the effectiveness of commonly used insecticides in apricot cultivation. The dose-response curves showed LC_50_ values ranging from 0.08 mg/L for flupyradifurone, 0.15 mg/L for acetamiprid, 0.70 mg/L for etofenprox, 1.89 mg/L for sulfoxaflor, 2.64 mg/L for pirimicarb, 3.97 mg/L for deltamethrin, up to 6.79 mg/L for tau-fluvalinate. These aforementioned insecticides resulted in mortality rates ranging from 95 to 100% at the field dose. Azadirachtin, flonicamid, and pyrethrins showed mortality rates of 27 to 45%. Spirotetramat reduced the colony development and decreased the number of infested shoots by 86%. Spinosad, which is not recommended against aphids, showed minimal impact; reducing the number of exuviae in nymphs in the colony development bioassay. It can be concluded that the majority of the tested insecticides are effective against *M. mumecola*.

## 1. Introduction

*Myzus mumecola* (Matsumura, 1917), also known as the apricot aphid, is an insect pest that affects apricots (*Prunus armeniaca* L.) and Japanese apricots (*Prunus mume* (Siebold)). It belongs to the Hemiptera order, Aphididae family, and Aphidinae subfamily [1]. Its secondary host plants are not yet definitively known [2].

Originally from Asian countries such as Japan, China, Taiwan, East Siberia, North-West Himalaya, and India [3,4,5], *M. mumecola* is currently spreading in Europe. This invasive aphid species was first discovered in Italy in 2016 [2,6], followed by Hungary in 2020 [7], Serbia in 2021 [3], and Germany in 2022 [8]. In South Tyrol, Italy, where this study was conducted, *M. mumecola* was identified for the first time in spring 2020. Since then, it has rapidly spread throughout the region becoming a significant threat to apricot trees.

Until now, aphids have not been considered as major pests of apricots in Europe and targeted control strategies against aphids have not been common. Indeed, only few species were known in Europe to create small colonies, sometimes overwintering on apricots: *Brachycaudus cardui*, *Brachycaudus hlichrysi*, *Hyalopterus pruni* [9], *Myzus persicae* [10], and *Phorodon humuli* [11,12]. In contrast to these already-established aphid species, it was observed that the infestation with *M. mumecola* causes severe damage to apricot trees: infested leaves become distorted and tightly rolled perpendicularly to the main vein, resulting in the formation of affected, distorted shoots [2]. In South Tyrol, we observed that less severely infested leaves showed deformations, which were visible until the leaves fell in autumn. In case of heavy infestation, the shoots showed a decreased growth and dieback of the tips was also observed. At very high infestation densities, the aphids also directly fed on fresh shoots, particularly causing severe damage to young trees. In the absence of ants, the secretion of honeydew led to the development of sooty mold on young fruits and leaves. In Hungary, the confirmation of *Plum pox virus* (PPV) detected in *M. mumecola* samples designated it as a significant vector of PPV due to its substantial prevalence across numerous surveyed apricot orchards [7]. Moreover, laboratory bioassays have demonstrated *M. mumecola* to possess a potential for PPV transmission, with a rate of 12% (compared to a *M. persicae* transmission rate of 24.4%) [5]. Considering the impact of *M. mumecola* on apricot trees, knowledge about the efficacy of available insecticides is urgently needed.

This study aims to validate the effects of insecticides commonly used for controlling insect populations in apricot orchards, along with some insecticides that are currently not labeled for application on apricots in Italy. All insecticides were tested at the field dose and those that had led to highest mortality rates were further investigated for their LC_50_ values. Since it is necessary to also assess the sublethal effects of the insecticides to precisely validate their efficacy [13], those that had not led to high mortality rates were tested on colony development. Furthermore, frequently used insecticides in Italy for controlling aphid populations in apricot cultivation were included in a field trial.

## 2. Materials and Methods

### 2.1. Aphid collection

In May 2022 and May 2023, colonies of *M. mumecola* were collected from untreated apricot trees of the Goldrich and Vinschger varieties in Piglon/Piccolongo, as well as from Goldrich trees in Göflan/Covelano, both located in South Tyrol, Italy. The trees were inspected for leaves exhibiting perpendicularly rolled-up margins and then examined for the presence of vital aphid colonies, characterized by the simultaneous absence of winged adult aphids and the presence of a small number of nymphs with wings. Shoots bearing leaves containing the desired aphid colonies were picked and carefully placed in transparent plastic containers (D33 × H12 × W33 cm; Ermetici Giostyle, Italy), previously lined with laboratory paper on the bottom. The collection took place in the morning hours and the aphid colonies remained about 5 h inside the closed plastic containers at 20 ± 1 °C under light.

### 2.2. Species Identification by Molecular Analysis

Sampled *M. mumecola* aphids were identified based on their morphological characteristics. Other aphid species found on apricot trees differ significantly from *M. mumecola* in terms of size and color [2,3,7,14]. Later, the accurate identification was confirmed through the cytochrome c oxidase subunit I (COI) barcode sequence analysis. Three aphids per sampling site were tested.

To extract the DNA from each of the six individual specimens, the samples were homogenized in 400 µL of cetyltrimethylammonium bromide (CTAB) buffer (CTAB 2.5%, Tris pH 8 100 mM, NaCl 1.4 M, EDTA 50 mM pH 8, PVP-40 1%, Proteinase K 10 mg/mL) using a microcentrifuge tube containing a 5 mm tungsten carbide bead (Qiagen, Hilden, Germany). The samples were disrupted using a Retsch Mixer Mill MM 400 at 30 Hz for 3 min. Following disruption, total DNA was extracted using the DNeasy Plant Mini Kit (Qiagen, Germany) according to the instructions provided. A specific region of the COI gene, known as a partial COI gene region, was amplified using the primers HCO2198 (5′GGTCAACAAATCATAAAGATATTGG3′) and LCO1490 (5′TAAACTTCAGGGTGACCAAAAAATCA3′) [15]. The thermal cycling conditions included an initial denaturation step of 5 min at 96 °C, followed by 4 cycles of which each one was composed of: 96 °C for 1 min, 47 °C for 1 min, and 72 °C for 1 min; then additional 35 cycles, each one implying 96 °C for 1 min, 50 °C for 1 min, and 72 °C for 1 min; and a final extension step of 72 °C for 5 min.

The generated amplicons were purified using the QIAquick PCR and Gel Cleanup Kit according to the manufacturer’s instructions (Qiagen, Germany). The purified amplicons were sequenced by LGC Genomics GmbH (Berlin, Germany).

To determine the identity of the obtained sequences, the integrated bioinformatics platform Barcode of Life Data System (BOLD System; http://www.barcodinglife.org, accessed on 20 July 2023) was utilized, and a nucleotide Basic Local Alignment Search Tool (BLAST) was conducted against the National Center for Biotechnology Information (NCBI) GenBank.

### 2.3. Insecticides

A total of 12 plant protection products were subjected to testing, each of them approved for integrated pest management in Italy (Table 1). The field doses for controlling aphids in apricot orchards were determined based on the instructions provided by the manufacturers, when available (http://www.fitosanitari.salute.gov.it/, accessed on 20 July 2023). Laser^TM^ and Trebon^®^ Up are not recommended to be used against aphids, therefore field doses for application against thrips in apricot cultivation were applied. Teppeki^®^ and Sivanto^®^ Prime are not approved for apricot production, therefore field doses for application against aphids in apple cultivation were applied.

Dose-response curves were generated for the most effective insecticides, namely acetamiprid, deltamethrin, flupyradifurone, pirimicarb, sulfoxaflor, and tau-fluvalinate. The LC_50_ values after 48 h were determined using a log_10_ series of 7 dilutions ranging from 0 mg/L to a maximum of 1000 mg/L of active ingredient. Less effective insecticides were evaluated for their effects on colony development.

### 2.4. Plant Material and Insecticide Application

Healthy shoots similar in age and size were collected from untreated apricot trees located in Piglon/Piccolongo (46°21′35.4″ N/11°17′04.7″ E), where the chosen trees of the Goldrich and Vinschger varieties showed evident *M. mumecola* infestation. Young shoots with leaves were cut off in the morning and transferred to the laboratory. The leaf-dip method was used to test the toxicity of the insecticides [16]: each insecticide was diluted in a 1 L beaker glass with 0.5 L distilled water, and the solution placed on a magnetic stirrer; collected shoots were cut into smaller pieces with three to five leaves each piece, then dipped completely in the insecticidal solution for 1 min, with a gentle agitation of 190 rpm; later, leaves were placed with the shoot end dipped in a 100 mL Erlenmeyer flask filled with tap water, and kept at room temperature for about 3 h to surface-dry. Leaves dipped into distilled water served as control.

### 2.5. Field Dose Bioassay and Dose-Response Curves

Once the leaves were completely surface-dry, 2 to 3 discs including the midrib were excised from each leaf with a cork borer (diameter = 14 mm). Each leaf disc was placed with the abaxial surface upwards in one well of a multi-well plate (24-well plates, flat bottom, diameter = 15.6 mm; VWR, Germany), previously filled with 1.2 mL water agar (15 g/L agar; Difco, Becton Dickinson, France). Only the wells positioned on the upper and lower edges of the plate were used, resulting in twelve leaf discs per plate overall.

**Figure 1:**
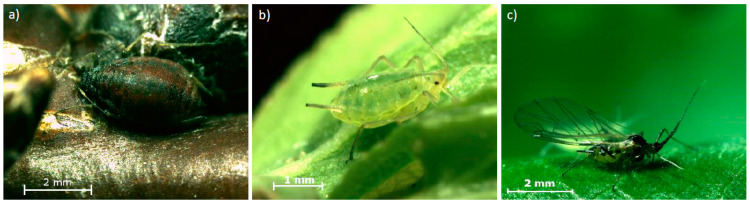
Adult stages of *M. mumecola* present on apricot trees in spring: (**a**) fundatrix, (**b**) apterae female, and (**c**) alatae female.

Only wingless female adult aphids were selected for the laboratory bioassays: these aphids are characterized by a clear green coloration that easily consent to distinguish them from the dark brown fundatrices (Figure 1). In addition, adult aphids were distinguished from nymphs on the basis of body size, distinct dark green lines, a reddish head, and an at the edges clearly upward bulged ovoid body. Aphids were gently picked using a fine, soft painting brush and transferred to the treated leaf discs within the wells. Only aphids that independently left the leaves and were walking on the container surfaces, showing healthy and active behavior, were taken. Once all the 12 aphids were transferred into the wells, the whole plate was covered with a taut transparent film, 10 holes per well were made with a needle for air circulation, and the plates were stored in a climate chamber at 20 ± 1 °C, 65 ± 5% relative humidity, under a 16:8 h L:D (light:dark) photoperiod. Plates were arranged in a completely randomized design, with one plate per replicate. Five replicates for the field dose bioassay and three replicates for the dose-response curves were performed. After 48 h, the numbers of dead and alive aphids were evaluated. The presence of nymphs and the absence of last instar exuviae showed that adult aphids were correctly distinguished from nymphs.

### 2.6. Colony Development Bioassay

Additional laboratory bioassays were conducted for insecticides that resulted in adult mortality rates of less than 75%, as measured using a colony development bioassay. After the application of insecticides through the leaf-dip method and waiting for surface-drying as described in Section 2.5, one leaf disc (diameter = 5 cm) including the midrib was cut out with a scalpel and placed with the abaxial surface upwards in a 6 cm Petri dish filled with water agar (15 g/L agar; Difco, Becton Dickinson, France). Aphids were collected and transferred as described in Section 2.5. After transferring one adult aphid per Petri dish, it was covered with a taut transparent film and 50 holes were made with a needle for air circulation. Single Petri dishes were used as replicates (*n* = 10). The Petri dishes were arranged as described in Section 2.5. After 72 h, the numbers of live nymphs, dead nymphs, deformed nymphs, and exuviae per adult aphid were evaluated. Only colonies with living adult aphids, which survived the 72 h experimental period were evaluated (ranging from *n* = 5 to 10).

### 2.7. Field Trial

The insecticides with active ingredients acetamiprid, azadirachtin, and spirotetramat were tested in a 7-year-old apricot orchard in Pfatten/Vadena (46°22′58.5″ N 11°17′26.2″ E) in 2023. The trials were conducted on scattered apricot trees of the variety Orange Red, grafted on the Wavit rootstock. Eight single trees, receiving the same agronomic and pest management practices, served as replicates. Treatments were arranged in a randomized complete block design. All trees showed on the 27 March the presence of fundatrices of *M. mumecola* (Figure 1a). Each tree was treated with a corresponding volume of 0.6 L insecticide solution, using an electric knapsack sprayer equipped with an anti-drift fan nozzle, CVI 110° green (Serena EL 16 LT; Italdifra Agricultural Tools S.r.l., Francofonte, Italy) at 2.5 bar. Applications were performed at the end of flowering (BBCH 72) on the 29 March. The application volume was chosen based on tree size to ensure complete coverage. The applied doses were established according to the manufacturer’s instructions (Table 1). Infestation was evaluated on the 27 April when the shoots had a length of approximately 30 cm (BBCH 75) and measured as the number of shoots from the current year bearing at least one colony of *M. mumecola* each. Meteorological data were obtained from the meteorological station present at the Laimburg Research Centre (46°22′56.8″ N, 11°17′19.5″ E).

### 2.8. Statistical Analyses

Analyses were performed using R version 4.2.1 (the R Foundation for statistical Computing, http://www.R-project.org, accessed on 20 July 2023). The mean mortality for the field dose was calculated as percentage of dead aphids per multi-well plate. The mortality data from the field dose bioassay were analyzed using a generalized linear model fitted with a Gaussian error distribution, followed by a Fisher’s least significant difference (LSD) test (*p* < 0.05).

Dose-response curves for the calculation of the half maximal lethal concentrations (LC_50_) were fitted with a four-parameter log-logistic function (LL.4) fixed to 0% and 100% corrected mortality (R software package ‘drc’, version 3.0-1 [17]). Abbott’s formula was used to calculate the corrected mortality data [18]:

$$Corrected\;mortality=\frac{(mortality\;in\;treatment\left[\%\right]-mortality\;in\;control\left[\%\right])\times 100}{\left(100\%-mortality\;in\;control\left[\%\right]\right)}.$$

Statistical significance of EC_50_ values was based on nonoverlap of 95% confidence intervals (*p* < 0.01).

Both the data from the colony development bioassay and the field trial were analyzed using a generalized linear model. Fisher’s least significant difference (LSD) test was performed independently for each variable (*p* < 0.05).

Figures were drawn using ‘Tidyverse’ (R software package ‘tidyverse’, version 2.0.0 [19]).

## 3. Results

### 3.1. Insect Identification

Three different adult stages of *M. mumecola* were present on the apricot trees throughout the spring. In the early season, the fundatrices, which hatch in early spring from the black overwintering eggs are feeding on the buds (Figure 1a). As soon as green tips are emerging, they produce green unwinged (apterae) females (Figure 1b), which migrate to the leaves where they start producing large colonies. At the end of spring (May–June), the apterae females give birth to winged (alatae) females (Figure 1c), which migrate to an unknown secondary host plant.

The BLAST conducted on sequences obtained from six adult aphids revealed a sequence identity of 99.84% with the deposited *M. mumecola* sequence (ID: MT635059.1), with a 100% query coverage and an E-value of 0.0. The BOLD Systems database confirmed a 100% similarity with *M. mumecola*. All six analyzed sequences present a 100% identity among themselves.

### 3.2. Adults’ Mortality Rates at Field Dose

**Figure 2:**
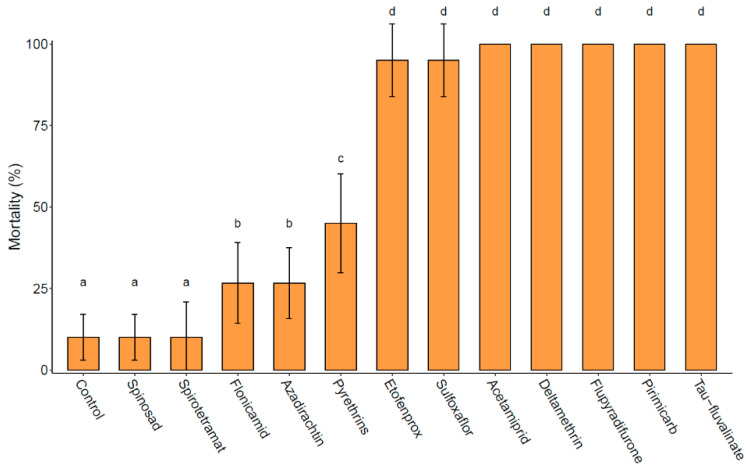
Mean (±SD) percent *M. mumecola* wingless adult mortality after 48 h of exposure to different insecticides at the field dose (*n* = 5). Means sharing the same letter are not significantly different (LSD test, *p* < 0.05).

The field dose bioassay revealed a significant impact on the mortality rate of *M. mumecola* adults feeding on treated leaf discs (F = 113.8; df = 12, 52; *p* < 0.001) (Figure 2). Among the tested insecticides, only spinosad and spirotetramat showed no significant effect on the mortality rate within 48 h. Both the flonicamid and azadirachtin treatments resulted in an average mortality rate of 27%, while the pyrethrins treatment led to a mortality rate of 45%. Etofenprox and sulfoxaflor exhibited a substantial impact with an average mortality rate of 95%, and their effects were not significantly different from those caused by acetamiprid, deltamethrin, flupyradifurone, pirimicarb, and tau-fluvalinate. In the latter cases, none of the adults survived throughout the experimental period.

### 3.3. Dose-Response Curves

**Figure 3:**
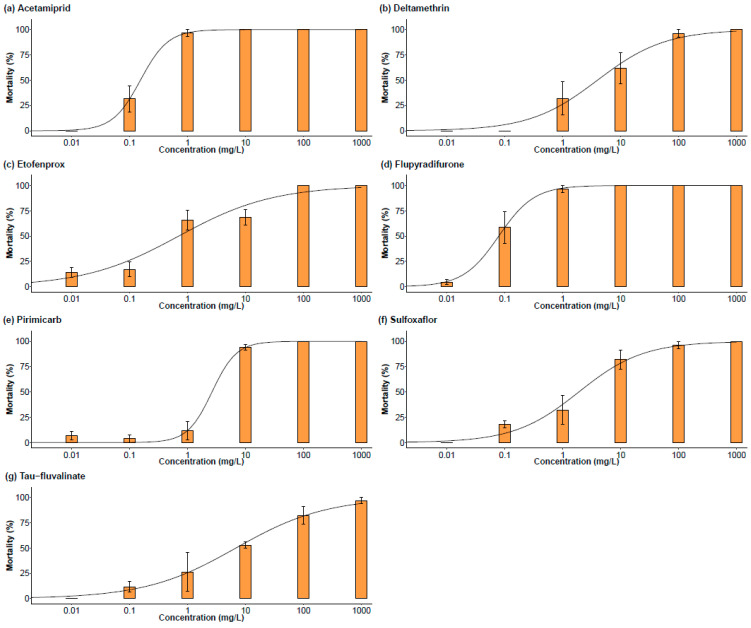
Mean (±SEM) percent *M. mumecola* wingless adult mortality after 48 h of exposure to different insecticides at 6 doses (0–1000 mg/L) (*n* = 3). Dose-response curves were fitted with a four-parameter log-logistic function (LL.4), resulting in different slopes: (**a**,**d**,**e**) steep and (**b**,**c**,**f**,**g**) slightly sloped. LC_50_ values are reported in Table 2.

Dose-response curves were conducted for seven insecticides that exhibited high mortality rates at the field dose (Figure 3). It can be observed that the sigmoid dose-response curves for acetamiprid, flupyradifurone, and pirimicarb are steep, while the dose-response curves for deltamethrin, etofenprox, sulfoxaflor, and tau-fluvalinate are slightly sloped.

The observed 95% confidence intervals for the LC_50_ values after 48 h after exposure varied significantly among the tested insecticides (Table 2). The observed LC_50_ values were 0.08 mg/L for flupyradifurone, 0.15 mg/L for acetamiprid, 0.70 mg/L for etofenprox, 1.89 mg/L for sulfoxaflor, 2.64 mg/L for pirimicarb, 3.97 mg/L for deltamethrin, and 6.79 mg/L for tau-fluvalinate. Flupyradifurone showed the lowest LC_50_ value, which was significantly more toxic than the other tested insecticides, except acetamiprid. The significant differences in LC_50_ values for flupyradifurone ranged from being 9-fold lower compared to etofenprox to 85-fold lower LC_50_ value compared to tau-fluvalinate. The LC_50_ value for acetamiprid was significantly lower, with reductions that ranged from 13 to 45-fold compared to the LC_50_ values observed for sulfoxaflor, pirimicarb, deltamethrin, and tau-fluvalinate. Furthermore, etofenprox showed a significantly lower LC_50_ value compared to pirimicarb and tau-fluvalinate, with a 4-fold and 10-fold lower LC_50_ value, respectively. The highest LC_50_ value was observed for tau-fluvalinate. No significant differences were found among the LC_50_ values of deltamethrin, etofenprox, and sulfoxaflor. Additionally, the LC_50_ values of sulfoxaflor, pirimicarb, deltamethrin, and tau-fluvalinate did not differ significantly amongst the group. In all control treatments, the maximum mortality was ≤16.67%.

### 3.4. Effects on Colony Development

**Figure 4:**
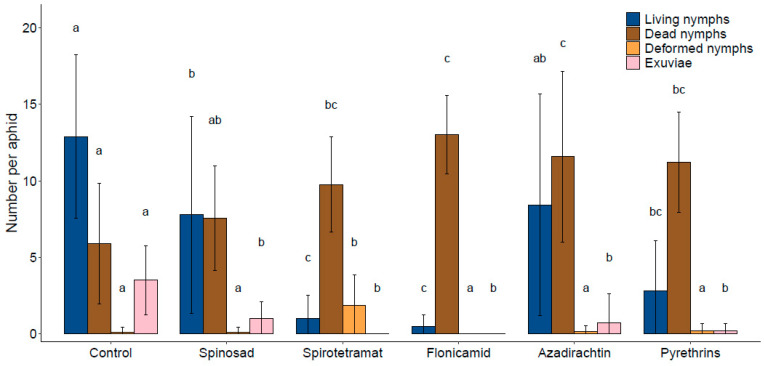
Mean (±SD) number of living nymphs, dead nymphs, deformed nymphs, and exuviae per aphid. Only data of aphids that survived the 72 h experimental period were included (ranging from *n* = 5 to 10). Means sharing the same letter are not significantly different between insecticide treatments (LSD test, *p* < 0.05).

The colony development bioassay revealed that, even when insecticide application at the field dose resulted in an adult mortality rate below 50%, noteworthy impacts on colony-forming capability were present (Figure 4).

While the insecticides did not result in significantly different total numbers of offspring per aphid (F = 2.32; df = 5, 41; *p* = 0.06), the newly formed colonies did exhibit a significant variation in the number of living nymphs (F = 8.83; df = 5, 41; *p* < 0.001), dead nymphs (F = 4.37; df = 5, 41; *p* = 0.003), deformed nymphs (F = 5.68; df = 5, 41; *p* < 0.001), and exuviae (F = 8.55; df = 5, 41; *p* < 0.001) per aphid.

Each insecticide was characterized by at least two significant impacts within the evaluated colony. Specifically, the lowest number of living nymphs was observed in the case of the spirotetramat and flonicamid treatments. Additionally, the spirotetramat colony showed the highest number of deformed nymphs, which are naturally unable to survive. The spirotetramat, flonicamid, azadirachtin, and pyrethrins treatments showed the highest comparable significative number of dead nymphs. Furthermore, all insecticide treatments led to a significant reduction in the number of exuviae, which serves as an indirect indicator for successful nymph development.

### 3.5. Field Efficacy

**Figure 5:**
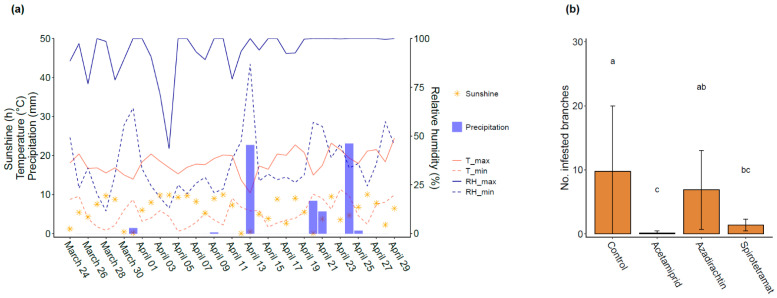
(**a**) Hours of sunshine, maximum and minimum relative humidity (RH), maximum and minimum temperature (T), and daily precipitation during the field trial time frame. (**b**) Mean (±SD, *n* = 8) number of *M. mumecola* infested shoots per tree on 27 April, after 30 days treatment with acetamiprid, azadirachtin and spirotetramat performed on 29 March. Means sharing the same letter are not significantly different (LSD test, *p* < 0.05).

During March and April 2023, the temperature and rainfall were characteristic for the weather of the region (Figure 5a). Most days were distinguished by sunshine and maximum temperatures of about 20 °C. Some very light rainfall was recorded after insecticide application.

The insecticides showed significant effects on *M. mumecola* infestation (F = 4.62; df = 3, 28; *p* = 0.009) (Figure 5b). The application of acetamiprid and spirotetramat resulted in a significant reduction in *M. mumecola* infested shoots in comparison to the unsprayed control. No significant differences in efficacy were observed between the control and the use of azadirachtin. Acetamiprid reduced the number of infested shoots by 98.7%, azadirachtin by 29.5%, and spirotetramat by 85.9%.

## 4. Discussion

Apricots are an important fruit crop in Europe: the total apricot yield in the European Union in 2022 was 540,000 tons, and Italy was amongst the top producers with 275,600 tons [20]. Therefore, the recent appearance of the invasive pest *M. mumecola* has raised legitimate concerns amongst producers. In 2016 in Italy’s Emilia-Romagna region, insecticides were applied in order to contain this pest; a significant percentage of the apricot orchards were treated, ranging from 60 to 70% of their total surface [2]. Therefore, it is necessary to provide up-to-date information about the efficacy of the available insecticides on the market.

In our region, both insecticides, acetamiprid and azadirachtin are an important part of strategies aimed at aphid control in apricot cultivation. Nevertheless, due to the variability of registered products between countries and over time, and considering the fact that the number of permitted active compounds within pest management control strategies is progressively decreasing, alternative insecticides with and without current usage approval on apricot were included in this study.

### 4.1. Adult Mortality Rates and Dose-Response Curves

Effective resistance management is essential to prevent the utility of current and future pesticides. Established in 1984, the Insecticide Resistance Action Committee (IRAC) classification scheme provides up-to-date information on the modes of action of insecticides and serves as the basis for developing appropriate insecticide resistance management strategies [21].

Among the tested plant protection products, the insecticides that resulted in a *M. mumecola* adult mortality rate of 95 to 100% at the field dose were the carbamate (IRAC group 1A) pirimicarb; the three pyrethroids (IRAC group 3A) deltamethrin, tau-fluvalinate, and etofenprox; the neonicotinoid (IRAC group 4A) acetamiprid; the sulfoximine (IRAC group 4C) sulfoxaflor; and the butenolide (IRAC group 4D) flupyradifurone.

By comparing the LC_50_ values of these insecticides to those obtained under similar conditions for *M. persicae*, an aphid species from the same genus, known as an established pest of various agricultural crops, should help to evaluate the obtained results.

Acetamiprid showed an LC_50_ value of 0.15 mg/L against *M. mumecola*, consistent with the reported values for *M. persicae*, which ranged from 0.017 to 1.42 mg/L [22,23]. Deltamethrin led to a LC_50_ value of 3.97 mg/L for *M. mumecola*, corresponding to the values reported for *M. persicae,* which range from 1.34 to 381 mg/L [16,24]. Etofenprox showed a LC_50_ value of 0.70 mg/L, while values reported for *M. persicae* were much lower and ranged between 0.026 to 0.089 mg/L [25]. Flupyradifurone showed a LC_50_ value of 0.08 mg/L for *M. mumecola*, while reports of the LC_50_ value for *M. persicae* ranged from 0.008 to 0.64 mg/L [23,26]. Pirimicarb showed a LC_50_ value of 2.64 mg/L, while the LC_50_ value for populations of *M. persicae* carrying different resistance levels was determined to be above 7.34 mg/L [27,28,29]. Sulfoxaflor showed a LC_50_ value of 1.89 mg/L, while the LC_50_ value of sulfoxaflor for *M. persicae* adults was determined to be between 0.001 and 48.9 mg/L [23,30,31]. Tau-fluvalinate led to a LC_50_ value of 6.79 mg/L, reported LC_50_ values of tau-fluvalinate for *M. persicae* adults ranged from 0.98 to 169 mg/L [24].

These findings showed that the effective dose for *M. mumecola* varies between insecticides, and they were similar to the dose reported for *M. persicae* in most cases. Since none of the LC_50_ values were very high, it can be assumed that the introduced *M. mumecola* population was not carrying any resistances against the seven tested insecticides. The results lead to the conclusion that the tested insecticides can be used to effectively control *M. mumecola*.

Among plant protection products tested in this study, insecticides that led to an adult mortality rate below 50% were the pyrethrins (IRAC group 3A) (45%), the flonicamids (IRAC group 29), and azadirachtin (IRAC unknown action) (27%). The spinosyn (IRAC group 5), spinosad, and the tetramic acid derivate (IRAC group 23), spirotetramat, showed no effect on adult mortality.

A comparison of the obtained results to those observed for *M. persicae* showed that: Pyrethrins, at the field dose of 29.78 mg/L, led to 45% adult mortality, while its 24 h LC_50_ value for *M. persicae* was reported to be between 0.007 and 62 mg/L [32,33]. Flonicamid, at the field dose of 70 mg/L, led to a 27% adult mortality, while *M. persicae* has a LC_50_ value of 0.10 mg/L [23]. Azadirachtin, at the field dose of 30 mg/L led to 27% mortality, while *M. persicae* has a much higher LC_50_ value of 393 mg/L [34]. Spinosad, at the field dose of 144 mg/L showed no effect on adult mortality, while on *M. persicae*, its 24 h LC_50_ values were determined to be 24.5 [35] and 35.7 mg/L [36]. Spirotetramat, at the field dose of 120 mg/L also showed no significant effect on adult mortality. This is likely because spirotetramat toxicity varies greatly among aphid species. Its LC_50_ value for *M. persicae* was found to be 0.17 mg/L [23].

### 4.2. Colony Development

Since the adult mortality rate for the insecticides azadirachtin, flonicamid, spinosad, spirotetramat, and pyrethrins was below 50%, the effects on the surviving adults and, potentially, on the development of the colony were further investigated. Azadirachtin led to fewer exuviae, indicating that nymph development was inhibited. This compound has an antifeedant effect, as well as growth-, fecundity-, and development-inhibiting effects on many insect pests [37]. However, the effects on the colony development of *M. mumecola* appears to be limited. Flonicamid treatment resulted in almost no living nymphs. Recent studies showed that while flonicamid induces high mortality of nymphs after a short period, starvation effects on adults were observed after a prolonged exposure [38,39]. Furthermore, considering the low adult mortality rate along with the higher efficacy against the nymphs, the action of flonicamid against *M. mumecola* might be based through the successful control of its offspring development. Pyrethrins resulted in an increased number of dead nymphs; this effect relies on inhibition of insects’ feeding capability [33]. Spirotetramat caused high nymph mortality and led to an increased number of deformed nymphs. This can be explained by the fact that spirotetramat led to deformations during egg development and has a high efficacy on immature aphids [40,41]. Spinosad only showed a reduced number of living nymphs and number of exuviae. It can be concluded that spinosad has an inhibitory effect on nymph development. These results are not surprising, considering that spinosad is not highly systemic and exhibits limited translaminar movement within plant leaf tissue [42]. Consequently, the insecticidal efficacy of spinosad against aphids is comparatively weaker than that observed for lepidopterans [43]. However, it was included in this study because it is frequently used in apricot cultivation against other insect pests.

### 4.3. Field Efficacy

The field trial showed that acetamiprid at the field dose is highly effective against *M. mumecola* by reducing the number of infested shoots by 99% within one month after treatment. Based on the results, it can be inferred that a post flowering treatment with acetamiprid is suitable to effectively control *M. mumecola*. This can be attributed to its rapid mode of action and mobility within plant tissues [44].

Additionally, spirotetramat has a significant impact on fecundity and population development under field conditions as evidenced by its high effectiveness in reducing infested shoots over time. Moreover, it is important to consider that for effectively controlling sucking insects, such as *M. mumecola*, hidden in plant leaves, phloem-mobile products like spirotetramat, which mainly act through ingestion, are considered desirable control options [45].

In the field trial, azadirachtin decreased the number of infested shoots by 20%; however, the result was not significantly different than the control. This suggests that currently registered dosages may need to be higher [46], and that the efficacy could potentially be increased by combining azadirachtin with other synergistic products, or alternatively using systemic application to avoid spraying runoff from leaves [46,47].

Suppression of aphids with alternative agronomic tools remains inconsistent; therefore, aphid pest control strategies generally rely on the use of insecticides. Further studies should focus on factors influencing the effectiveness of insecticides, such as the phenological stage of the plant and aphid population density and life cycle. Furthermore, integrating non-chemical control measures with these findings could contribute to effective integrated pest management strategies. Additionally, it is important to expand surveys on natural enemies that are present on infested apricot trees, such as coccinellids, hoverflies, chrysopids, parasitoids, and entomopathogenic fungi [6]. Integrating these aspects into future studies will contribute to a more comprehensive understanding of biological control options. Given the potential for aphids to evolve insecticide resistance and the variability in insecticide sensitivity across aphid populations, it is necessary to conduct a future assessment for *M. mumecola* populations’ responses to insecticides over time, considering the variation in natural populations from diverse geographical areas. Moreover, developing integrated pest management strategies involves identifying the economic threshold level of *M. mumecola*.

## 5. Conclusions

The introduction of *M. mumecola* leads to the need to adapt plant protection strategies against aphids in apricot cultivation. The results of this study provide data on the efficacy of insecticides to control *M. mumecola*. Among conventional insecticides, the most effective were acetamiprid, deltamethrin, etofenprox, flupyradifurone, pirimicarb, sulfoxaflor, and tau-fluvalinate. Deltamethrin, tau-fluvalinate, and etofenprox belong to the same IRAC group (3A). Furthermore, spirotetramat also showed a good efficacy in the field trial. However, it is worth noting that the approval for the use of spirotetramat is scheduled to end in 2025 (Commission Implementing Regulation (EU) 2022/489).

Regarding organic insecticides, pyrethrins showed a significant effect on adults and nymphs, while azadirachtin was less effective. Spinosad, which is also allowed in organic production but not recommended for aphids, showed low efficacy; therefore, no effect on *M. mumecola* can be expected. Based on the results, it can be concluded that successful control of *M. mumecola* is possible with synthetic chemical insecticides, while insecticides allowed for organic production were less effective in achieving the same level of control.

## Figures and Tables

**Table 1:** List of insecticides included in the laboratory bioassays and field trial; their active ingredient (AI), trade name, Italian manufacturer/distributor, composition and the applied field dose.

Active Ingredient	Trade Name	Manufacturer/Distributor	Composition (AI)	Used Field Dose (AI)
Acetamiprid	Epik^®^ SL	Sipcam Italia S.p.A.	50 g/L	100 mg/L
Azadirachtin	NeemAzal^®^-T/S	Biogard, CBC (Europe) S.r.l.	10 g/L	30 mg/L
Deltamethrin	Decis^®^ Evo	Bayer CropScience S.r.l.	25 g/L	12.5 mg/L
Etofenprox	Trebon^®^ Up ^a^	Sipcam Italia S.p.A.	287.5 g/L	143.75 mg/L
Flonicamid	Teppeki^® b^	Belchim Crop Protection Italia S.p.A.	500 g/kg	70 mg/L
Flupyradifurone	Sivanto^®^ Prime ^b^	Bayer CropScience S.r.l.	200 g/L	150 mg/L
Pirimicarb	Pirimor^®^ 50	ADAM Italia S.r.l.	500 g/kg	375 mg/L
Pyrethrins	Biopiren Plus^®^	Biogard, CBC (Europe) S.r.l.	18.61 g/L	29.776 mg/L
Spinosad	Laser^TM a^	Corteva Agriscience Italia S.r.l.	480 g/L	144 mg/L
Spirotetramat	Movento 48 SC	Bayer CropScience S.r.l.	48 g/L	120 mg/L
Sulfoxaflor	Closer^TM^	Corteva Agriscience Italia S.r.l.	120 g/L	36 mg/L
Tau-fluvalinate	Mavrik^®^ Smart	ADAMA Italia S.r.l.	240 g/L	288 mg/L

^a^ Currently not labelled against aphids in Italy. ^b^ Currently not labelled on apricot in Italy.

**Table 2:** Adulticide effect of insecticides applied on apricot leaf discs against *M. mumecola* adults after 48 h exposure.

Active Ingredient	LC_50_^α^ (mg/L)	CI 95% ^β^ (mg/L)	Slope (±SE)	df ^γ^	χ^2^
Acetamiprid	0.15	0.10–0.21 ^ab^	1.82 (±0.60)	19	47.73
Deltamethrin	3.97	1.13–6.82 ^cd^	0.75 (±0.14)	19	58.80
Etofenprox	0.70	0.19–1.22 ^bc^	0.53 (±0.09)	19	77.00
Flupyradifurone	0.08	0.05–0.10 ^a^	1.42 (±0.43)	19	51.23
Pirimicarb	2.64	1.65–3.62 ^d^	2.07 (±0.35)	19	70.33
Sulfoxaflor	1.89	0.83–2.95 ^cd^	0.78 (±0.16)	19	79.33
Tau-fluvalinate	6.79	1.58–12.01 ^d^	0.56 (±0.10)	19	74.66

^α^ LC_50_: lethal concentration that is expected to cause 50% mortality. Different letters indicate significant differences (CI 95% did not overlap, *p* < 0.01). ^β^ CI 95%: confidence interval limits at 95%. ^γ^ df: degree of freedom.

## Data Availability

The data presented in this study are available from the corresponding author, upon reasonable request.
